# *IDH1* or -*2* mutations do not predict outcome and do not cause loss of 5-hydroxymethylcytosine or altered histone modifications in central chondrosarcomas

**DOI:** 10.1186/s13569-017-0074-6

**Published:** 2017-05-04

**Authors:** Arjen H. G. Cleven, Johnny Suijker, Georgios Agrogiannis, Inge H. Briaire-de Bruijn, Norma Frizzell, Attje S. Hoekstra, Pauline M. Wijers-Koster, Anne-Marie Cleton-Jansen, Judith V. M. G. Bovée

**Affiliations:** 1 Department of Pathology, Leiden University Medical Center, L1-Q, P.O. Box 9600, 2300 RC Leiden, The Netherlands; 2 1st Department of Pathology, Laikon General Hospital, Athens University School of Medicine, Athens, Greece; 3 Department of Pharmacology, Physiology & Neuroscience, School of Medicine, University of South Carolina, Columbia, USA; 4 Department of Human Genetics, Leiden University Medical Center, Leiden, The Netherlands

**Keywords:** 5-Hydroxymethylcytosine, 5-Methylcytosine, Histone methylation, Chondrosarcoma, Isocitrate dehydrogenase, Bone tumour, Enchondroma

## Abstract

**Background:**

Mutations in *isocitrate dehydrogenase* (*IDH)1* or -*2* are found in ~50% of conventional central chondrosarcomas and in up to 87% of their assumed benign precursors enchondromas. The mutant enzyme acquires the activity to convert α-ketoglutarate into the oncometabolite d-2-hydroxyglutarate (d-2-HG), which competitively inhibits α-ketoglutarate dependent enzymes such as histone- and DNA demethylases.

**Methods:**

We therefore evaluated the effect of *IDH1* or -*2* mutations on histone modifications (H3K4me3, H3K9me3 and H3K27me3), chromatin remodeler ATRX expression, DNA modifications (5-hmC and 5-mC), and TET1 subcellular localization in a genotyped cohort (*IDH*, succinate dehydrogenase (*SDH*) and fumarate hydratase (*FH*)) of enchondromas and central chondrosarcomas (n = 101) using immunohistochemistry.

**Results:**

*IDH1* or -*2* mutations were found in 60.8% of the central cartilaginous tumours, while mutations in *FH* and *SDH* were absent. The mutation status did not correlate with outcome. Chondrosarcomas are strongly positive for the histone modifications H3K4me3, H3K9me3 and H3K27me3, which was independent of the *IDH1* or -*2* mutation status. Two out of 36 chondrosarcomas (5.6%) show complete loss of ATRX. Levels of 5-hmC and 5-mC are highly variable in central cartilaginous tumours and are not associated with mutation status. In tumours with loss of 5-hmC, expression of TET1 was more prominent in the cytoplasm than the nucleus (p = 0.0001).

**Conclusions:**

In summary, in central chondrosarcoma *IDH1* or -*2* mutations do not affect immunohistochemical levels of 5-hmC, 5mC, trimethylation of H3K4, -K9 and K27 and outcome, as compared to wildtype.

**Electronic supplementary material:**

The online version of this article (doi:10.1186/s13569-017-0074-6) contains supplementary material, which is available to authorized users.

## Background

**Fig. 1:**
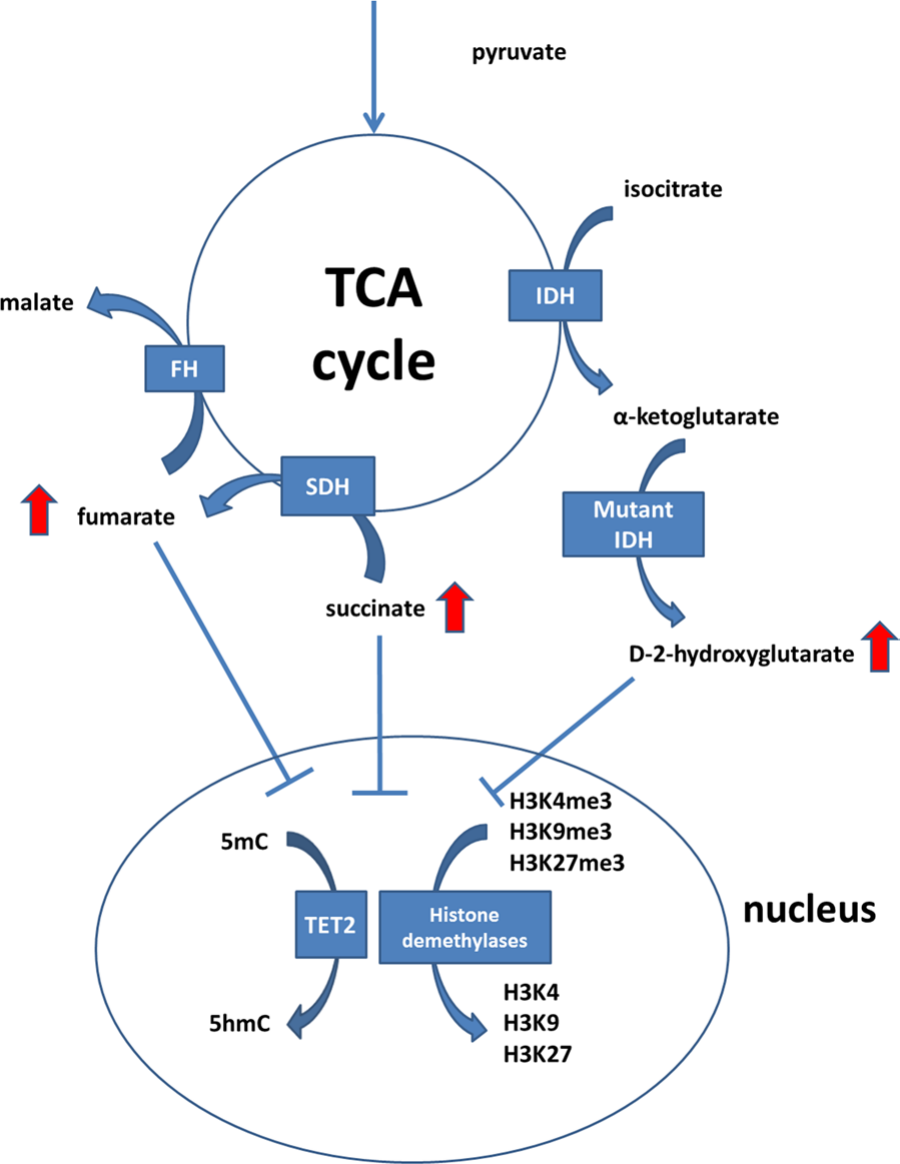
Schematic representation of the effect of the mutations in metabolic enzymes IDH, SDH, and FH on histone modification and DNA methylation. Accumulation of d-2-hydroxyglutarate (d-2-HG), succinate and fumarate competitively inhibit TET2, which converts 5-mC to 5-hmC, which is anticipated to cause loss of 5-hmC and global CpG island hypermethylation. In addition, competitive inhibition of histone demethylases causes altered histone modification

Mutations in *isocitrate dehydrogenase (IDH)*-*1* or -*2* (which we commonly refer to as *IDH*) are found in gliomas (60–80% [1, 2], acute myeloid leukemia (~20%) [3], cholangiocarcinomas (7–28%) [4–6] and in benign and malignant central cartilaginous tumours [7–9]. Isocitrate dehydrogenase (IDH) is an enzyme involved in the conversion of isocitrate to α-ketoglutarate in the tricarboxylic acid (TCA) cycle (Fig. 1). Mutations are exclusively found on the arginine residues R132 of *IDH1* and in R140 and R172 of *IDH2*.

Cartilaginous neoplasias affect the bone and can be roughly divided into benign (enchondromas and osteochondromas), and malignant (chondrosarcomas of different subtypes) [10]. Enchondromas occur in the medulla of bone [10], and occasionally they can present with multiple lesions in non-hereditary Ollier disease or Maffucci syndrome [11]. Conventional central chondrosarcoma is the most common chondrosarcoma subtype, affecting the medulla of bone, which can arise from a pre-existing benign enchondroma. Peripheral conventional chondrosarcoma affects the surface of the bone and arises from a preexisting benign osteochondroma. The best prognostic marker for chondrosarcoma so far is histological grading, since atypical cartilaginous tumour/grade I chondrosarcomas behave locally aggressive and only metastasize in exceptional cases. Grade II and III chondrosarcomas are more cellular and the chance of metastasis is increased (up to 70% of the patients for grade III) [12, 13].

Mutations in *IDH* are found in ~50% (38–86%) of conventional central chondrosarcomas, in ~54% of dedifferentiated and ~15% of periosteal chondrosarcoma, and in up to 87% of benign enchondromas [7–9], while being absent in peripheral, mesenchymal and clear cell chondrosarcoma. Patients with Ollier disease and Maffucci syndrome carry *IDH* mutations in a somatic mosaic fashion [8, 9]. While in gliomas the *IDH* mutations are associated with a more favorable outcome [2, 14, 15], in intrahepatic cholangiocarcinoma, as well as in leukemia, its prognostic value could not unequivocally be shown [16, 17].

The mutant enzyme acquires the ability to convert α-ketoglutarate into the oncometabolite d-2-hydroxyglutarate (d-2-HG), which shows structural similarities with α-ketoglutarate. Indeed, increased levels of d-2-HG have been found in cartilage tumours with an *IDH* mutation [8]. Elevated levels of d-2-HG were shown to competitively inhibit α-ketoglutarate dependent enzymes such as histone demethylases [18] (Fig. 1), specifically affecting trimethylation of the transcriptionally permissive histone mark H3K4, and the repressive histone marks H3K9 and H3K27 [19–21]. In addition, d-2-HG inhibits the ten-eleven translocation (TET) enzymes [18] (Fig. 1). TET enzymes are involved in DNA demethylation by catalyzing the conversion of 5-methylcytosine (5-mC) into 5-hydroxymethylcytosine (5-hmC) [22, 23]. Indeed, methylation arrays confirmed global hypermethylation in *IDH1* mutant enchondromas [9], and decreased levels of 5-hmC were detected in myeloproliferative disorders harboring *TET2* mutations [24]. Furthermore, nuclear exclusion of TET1 was shown to be related to loss of 5-hmC in gliomas without *IDH* mutations [25]. Wiestler et al. described that loss of ATRX (an ATP-dependent helicase that belongs to the SWI/SNF family of chromatin remodeling proteins and facilitates access to nucleosomal DNA) was almost exclusively found in gliomas harboring *IDH* mutations [26]. Germline mutations in *ATRX* are associated with X-linked intellectual disability with alpha-thalassemia (ATRX) syndrome, and generally cause loss of protein expression [27].

Other TCA cycle enzymes involved in cancer are succinate dehydrogenase (*SDH*) in hereditary paragangliomas [28–31] and gastrointestinal stromal tumour (GIST) [32], and fumarate hydratase (*FH*) in hereditary leiomyomas and renal cell cancer (HLRCC) [33]. Mutations in either *SDH* or *FH* lead to loss of function, resulting in accumulation of succinate and fumarate, respectively. Similar to d-2-HG, elevated levels of succinate and fumarate inhibit α-ketoglutarate dependent enzymes [34–36] (Fig. 1). In *SDH* mutant paragangliomas and GIST, and in *FH* deficient smooth muscle tumours, 5-hmC was low to absent [37, 38].

In this study, our aim was to evaluate the effect of *IDH* mutations on outcome, and on histone modifications (H3K4me3, H3K9me3 and H3K27me3), DNA modifications (5-hmC and 5-mC), chromatin remodeling (ATRX), and subcellular localization of TET1 in a cohort of enchondromas and central chondrosarcomas for which we determined mutation status of *IDH*, *SDH* and *FH*.

## Methods

### Patient series [tissue microarray (TMA)]

The first cohort of cartilaginous tumours used for the tissue microarray (TMA) has been described previously [39] and includes nine enchondromas, 11 osteochondromas, 92 central chondrosarcomas (of which 42 atypical cartilaginous tumours/grade I; 36 grade II and 14 grade III) and 45 peripheral chondrosarcomas (of which 31 atypical cartilaginous tumours/grade I; 11 grade II and 3 grade III). Thus, this TMA contains a cohort of 101 central cartilaginous tumours. Cores from skin, colon, tonsil, prostate, breast carcinoma, spleen and liver were included for control and orientation purposes.

A second cohort of enchondromatosis related tumours was used for which details were also described previously [40]. This second TMA was exclusively used for 5mC and 5hmC immunohistochemistry as a second separate independent series, and was analyzed in the same way as the first cohort. In total, the TMA contains cores from 86 tumours, of which 65 were enchondromatosis related (51 Ollier disease, 13 Maffucci syndrome, 1 polyostotic chondrosarcoma) and 21 solitary enchondromas and chondrosarcomas. Cores from growth plate, articular cartilage, breast carcinoma, prostate, colon, skin and tonsil were included for control and orientation purposes.

Whole sections derived from normal articular cartilage (n = 3) and growth plate (n = 3) were also taken along as controls. All controls were acquired from pathological resections unrelated to cartilaginous tumours. All samples were handled in a coded manner according to the ethical guidelines as described in the Code for Proper Secondary Use of Human Tissue in The Netherlands of the Dutch Federation of Medical Scientific Societies. Follow-up data were available for all subjects (range 7–344 months, mean 150, 9 months).

### Analysis of *IDH*, *SDH* and *FH* mutation status

Immunohistochemistry to detect the *IDH1* R132H mutation was previously reported for the first cohort and 6 out of 101 central cartilaginous tumours were shown to be positive [9]. For the 95 cases that were negative, we isolated the corresponding DNA. When available, we used frozen tissue, otherwise DNA was isolated from formalin-fixed, paraffin embedded tissue (FFPE). DNA isolation from fresh frozen material was performed using the wizard genomic DNA purification kit (Promega, Madison, WI) according to the manufacturer’s instructions. DNA isolation from paraffin embedded tissue was performed as described [41]. Hydrolysis probes assay for *IDH1* R132C and R132H was performed as described previously [9]. Subsequently, PCR amplification of exon 4 of *IDH1* was performed for 76 samples that were negative in the hydrolysis probes assay. After each PCR run, melting curves were inspected in order to check the formation of a single product. The PCR products were purified using the QIAquick PCR Purification Kit (Qiagen, Hilden, Germany) according to the manufacturer’s manual and Sanger sequencing was performed, as described previously [9]. For the remaining 64 samples without an *IDH1* mutation, exon 4 of *IDH2* was amplified and sequenced for mutations. Oligonucleotide sequences for PCR are shown in Additional file 1: Table S1. Bidirectional Sanger sequencing was performed by Macrogen Europe (Amsterdam, The Netherlands). The sequence results were evaluated using Mutation Surveyor software (Soft-Genetics). To exclude that *IDH* wildtype cartilage tumours harbor mutations in *SDH* or *FH*, TMAs were stained for SDHB (Atlas antibodies, HPA002868) and S-2-succinocysteine (2-SC) [42, 43] as described [37]. Mutations in SDH subunits destabilize the complex, which leads to degradation and loss of staining for SDHB, which is therefore widely used as a marker to screen for mutations in the SDH subunits [44]. Mutations in *FH* lead to accumulation of fumarate leading to aberrant succination of proteins. Positive staining for (S)-2-succinocysteine (2SC) was shown to be a reliable marker to screen for mutations in *FH* [42, 43].

### Immunohistochemistry

Immunohistochemistry was performed as described previously [37], for histone modifications (H3K4me3; Millipore, 07-473; H3K9me3, Abcam, ab8898; H3K27me3, Millipore, 07-449); a chromatin remodeler (ATRX; Sigma, HPA001906); DNA modifications (5-hmC; Active Motif, 39,769 and 5-mC; Millipore, 33D3) as well as for TET1 (Genetex, N3C1).

### Evaluation and scoring of immunohistochemistry

All slides were evaluated by two observers (JVMGB, JS, AHGC, GA in variable combination) independently and discrepancies were discussed to reach consensus. Scoring for both intensity (0 = negative, 1 = weak, 2 = moderate, 3 = strong) and the percentage of positive tumour cells (0 = 0%, 1 = <25%, 2 = 25–50%, 3 = 50–75%, 4 = 75–100%) were added up to a total sum score [45]. The average was taken of the three different cores for further analysis. Since nuclear exclusion of TET1 was associated with loss of 5-hmC in *IDH* wildtype gliomas, we specifically scored the subcellular localization of TET1 (N = nuclear, C = cytoplasmic, N>C=more nuclear staining than cytoplasmic, C>N=more cytoplasmic staining than nuclear) as described [25].

### Statistical analysis

Statistical significance between groups was determined using ANOVA with Tukey as posthoc analysis and Chi square test as appropriate. Kaplan–Meier curves were plotted for the different stainings to determine the effect on overall survival and metastasis free survival, statistical significance was determined using Log Rank test. Data was analyzed using the IBM SPSS Statistics 23 software.

## Results

### *IDH* mutations in 60.8% of central cartilaginous tumours

**Fig. 2:**
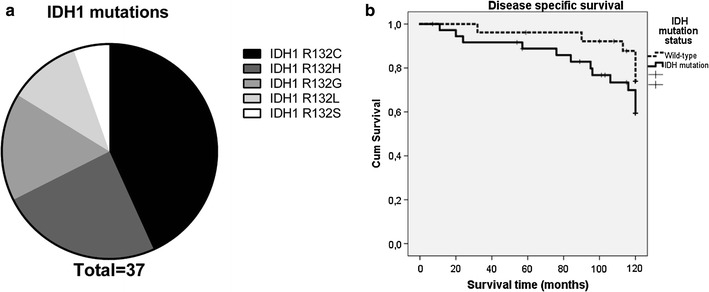
Genotyping of central cartilaginous tumours. **a** The mutational spectrum for *IDH1* in the central chondrosarcomas from the TMA. In total 37 samples harbored an *IDH1* mutation, of which the most prevalent mutation is the R132C. Eight samples harbored an *IDH2* R172S mutation. **b** Disease specific survival of patients with *IDH* mutated (R132C, R132H, R132G, R132l, R132S, R172S) chondrosarcomas compared to patients with wild-type *IDH* chondrosarcomas revealed no statistical significant difference (p = 0.183)

In total, the *IDH* mutation status could be determined for 74 out of 101 central cartilaginous tumours of the first cohort. Thirty-seven tumours contained a mutation in *IDH1* at the R132 position, for which the mutational spectrum is shown in Fig. 2a. In addition, eight samples harbored an *IDH2* R172S mutation. Furthermore, 27 tumours were confirmed to lack *IDH* hotspot mutations, two of which contained an *IDH1* G105G polymorphism. These two cases were excluded from analysis since this polymorphism was recently suggested to be a possible prognostic marker in leukemia [46]. Mutation analysis failed for 27 samples due to poor quality of the DNA derived from decalcified FFPE tissue. The clinicopathological data of the genetically confirmed *IDH* mutant versus wildtype patients are shown in Table 1.

**Table 1:** Clinicopathological data of the *IDH* mutant versus wild-type group

	*IDH* wild-type (n = 29)	*IDH* mutation (n = 45)
Male	9 (31%)	19 (42%)
Median age at diagnosis	51 (21–79)	51 (22–85)
Histology		
Enchondroma	1 (15%)	6 (85%)
ACT/grade I	14 (50%)	14 (50%)
Grade II	9 (34%)	18 (66%)
Grade III	5 (42%)	7 (58%)
Metastasis	4 (45%)	5 (55%)
Median follow-up (months)	137 (7–278)	121 (11–312)

*ACT* atypical cartilaginous tumour

### No association between *IDH* mutation and outcome

There was no significant difference in disease specific survival between *IDH* mutant and *IDH* wildtype chondrosarcomas in the first cohort (p = 0.183, Fig. 2b), independent from grade (data not shown). Also, when the different mutations were analyzed separately (R132C, R132H, R132G, R132l, R132S, R172S and wild-type) or in combination (the most common R132C and R132G versus the others) no difference in outcome was found (Additional file 2: Figure S1, p = 0.726), although numbers are small. Likewise, metastasis free survival was not associated with *IDH* mutation status (p = 0.96, data not shown).

### *SDH* and *FH* mutations were absent in chondrosarcoma

We excluded mutations in other metabolic enzymes in the 27 cartilaginous tumours that were wildtype for *IDH*, since all tumours were positive for SDHB immunohistochemical staining, suggesting an intact SDH complex and thereby excluding mutations in the different SDH subunits [44] (Additional file 3: Figure S2A; Additional file 4: Table S2). Furthermore, all primary tumours were negative for the presence of 2-SC (Additional file 3: Figure S2B; Additional file 4: Table S2), which was shown to be a robust biomarker for mutations in *FH* [42, 43].

### Trimethylation of H3K4, H3K9 and H3K27 was highly abundant in chondrosarcoma

**Fig. 3:**
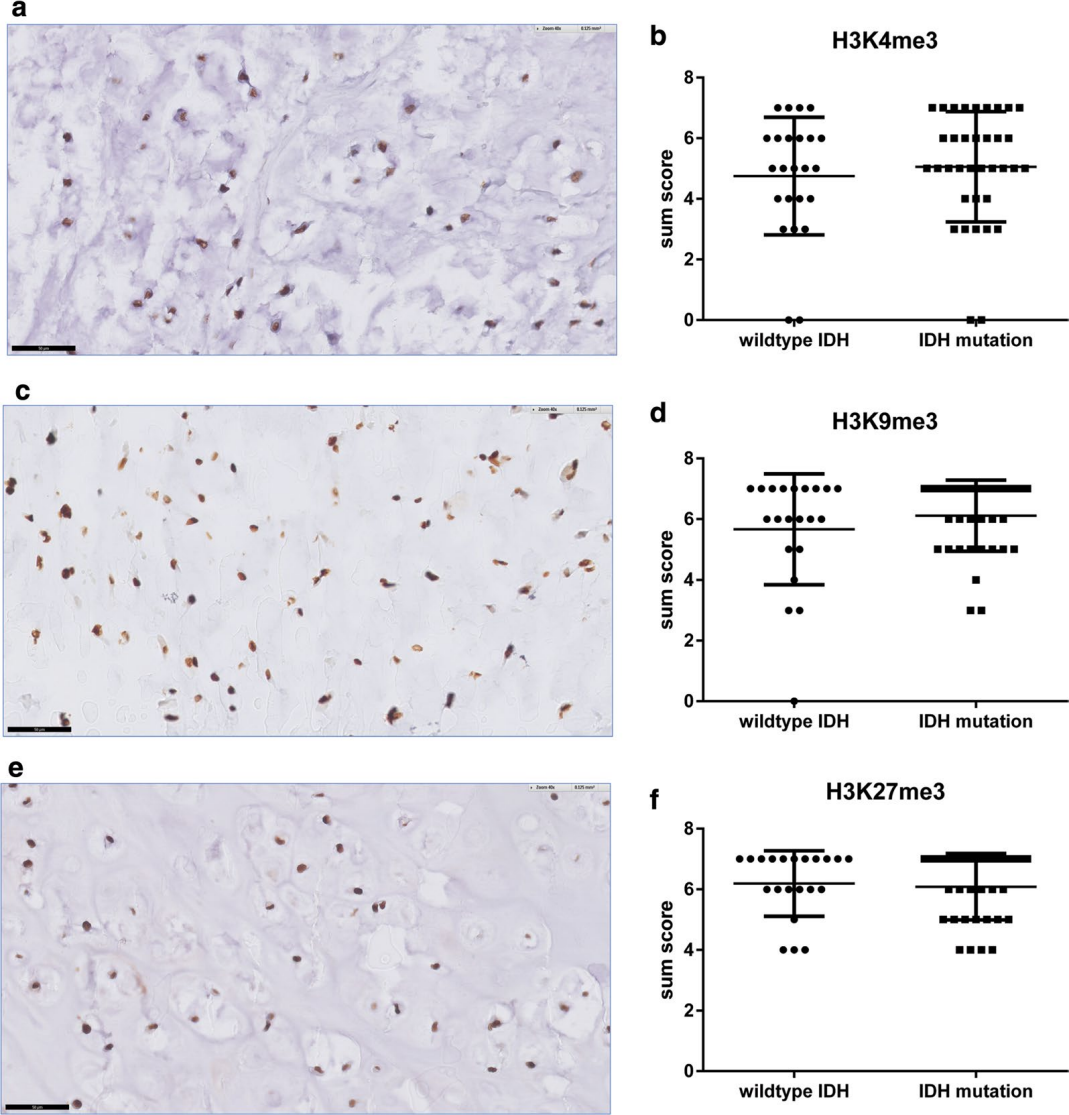
No difference in trimethylation of histone marks H3K4, H3K9 and H3K27 in central chondrosarcomas. Strong nuclear positivity for H3K4me3 (**a**), H3K9me3 (**c**) and H3K27me3 (**e**) in *IDH* wildtype central chondrosarcomas as well as central chondrosarcomas harboring an *IDH* mutation. Staining was scored as the sum of the intensity and the percentage of positive tumour cells (**b**, **d**, **f**). (Scores were rounded to zero decimal places and *black bar* indicates 50 µm)

The histone modification marks H3K4me3, H3K9me3 and H3K27me3 were abundantly expressed in the vast majority of central chondrosarcomas in the first cohort (Fig. 3a, c, e). Therefore, no difference in the levels of trimethylated H3K4, H3K9 or H3K27 could be detected between *IDH* mutant and wildtype central cartilaginous tumours (Fig. 3b, d, f, p = 0.54, 0.46 and 0.78, respectively), nor between central and peripheral chondrosarcomas (data not shown). Furthermore, studying the low grade and high grade central chondrosarcomas in separate groups revealed no significant differences, and there was no correlation with histological grade (p = 0.24, 0.51 and 0.89, respectively) (data not shown). Also, levels of trimethylation of H3K4, H3K9 and H3K27 were not associated with overall or metastasis free survival (data not shown).

### Loss of ATRX in 5.6% of conventional central chondrosarcomas

Since in gliomas loss of the chromatin remodeler ATRX can be found in *IDH* mutant tumours [26], we evaluated a possible co-occurrence in chondrosarcoma. Two out of 36 (5.6%) conventional chondrosarcomas in the first cohort for which staining was evaluable show complete loss of ATRX (data not shown). One of these two tumours harbored an *IDH1* R132 mutation, whereas the other tumour was confirmed to be wildtype for *IDH*.

### Variable levels of 5-hydroxymethylcytosine (5-hmC) and 5-methylcytosine (5-mC) in chondrosarcomas

**Fig. 4:**
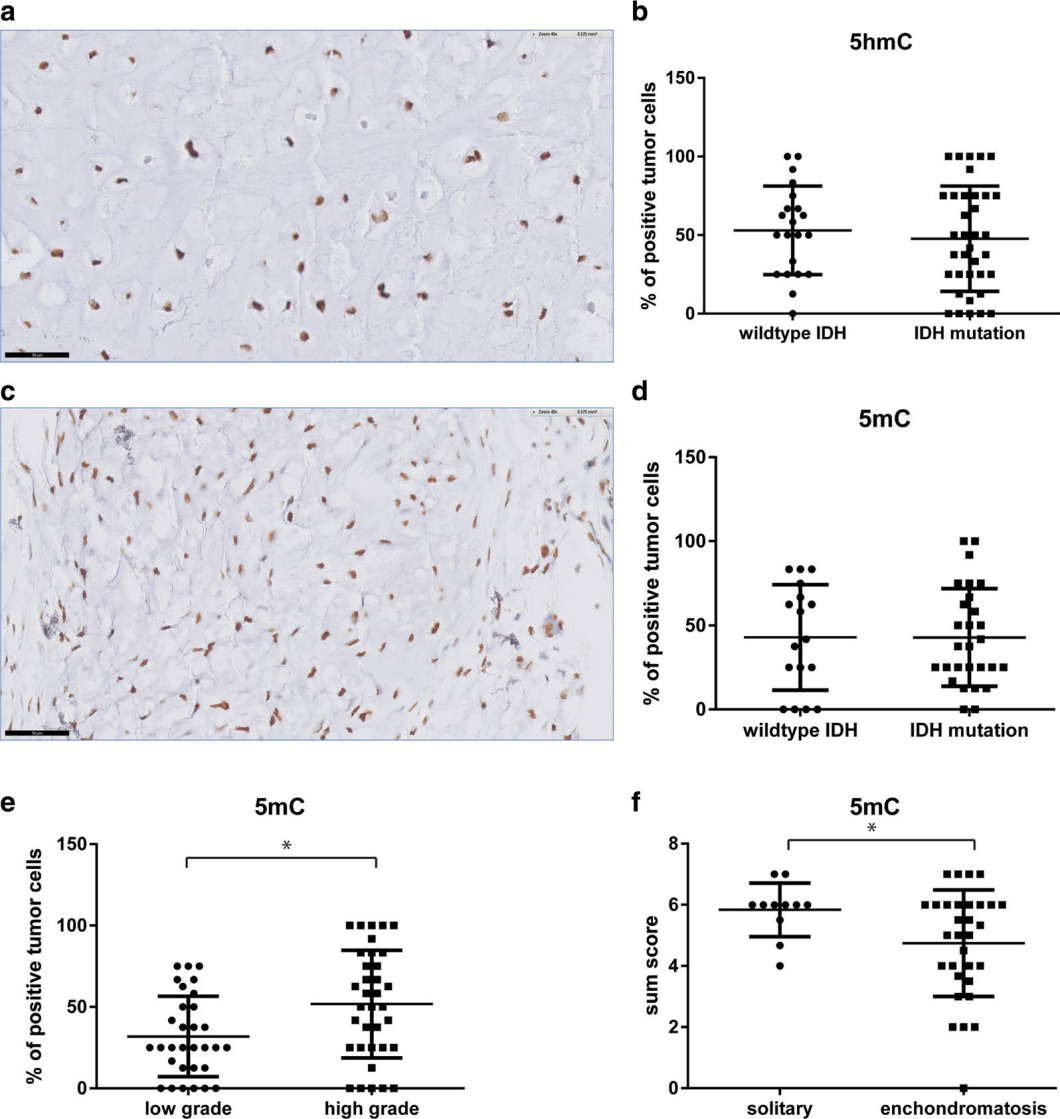
Variable levels of 5-hydroxymethylcytosine (5-hmC) and 5-methylcytosine (5-mC) in chondrosarcomas. Percentage of tumour cells positive for 5hmC (**a**, **b**) and 5mC (**c**, **d**) in *IDH* mutant versus *IDH* wildtype tumours (*black bar* indicates 50 µm). The percentage of 5-mC positive tumour cells was significantly higher in high grade chondrosarcomas as compared to low grade, which was independent of the *IDH* mutation status (**e**). Solitary chondrosarcomas showed significantly higher levels of 5mC compared to enchondromatosis related chondrosarcomas, independent of the histological grade (**f**)

**Fig. 5:**
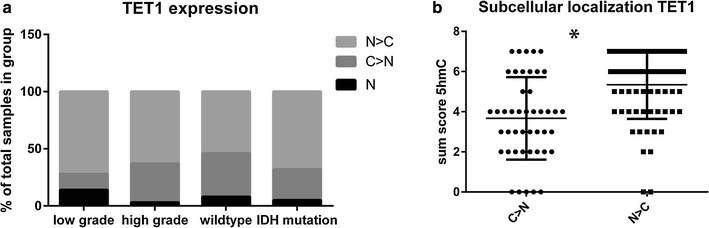
Lower 5hmC levels in tumours with decreased nuclear staining for TET1. **a** There is no significant difference in subcellular localization of TET1 in *IDH* mutant versus *IDH* wildtype chondrosarcomas, and complete nuclear exclusion of TET1 was absent. **b** In tumours in which the staining was predominantly cytoplasmic, 5-hmC levels were significantly lower as compared to tumours in which staining was predominantly nuclear (scores were rounded to zero decimal places)

Since d-2-HG competitively inhibits the TET enzymes [18], which catalyze the conversion of 5-mC into 5-hmC, we evaluated the distribution of 5-mC as well as 5-hmC. Levels of 5-hmC (Fig. 4a) and 5-mC (Fig. 4c) were highly variable in chondrosarcoma. In most tumours only a fraction of the tumour cells were positive. However, no significant difference could be observed in the levels of 5-hmC (Fig. 4b) or 5-mC (Fig. 4d) between *IDH* wildtype and *IDH* mutant chondrosarcomas, considering sum score, or considering intensity and percentage of positive tumour cells separately. Interestingly, in high grade chondrosarcomas the percentage of 5-mC positive tumour cells was significantly higher as compared to low grade chondrosarcomas, which was independent of the *IDH* mutation status (p = 0.013) (Fig. 4e). This was however not reflected by a detectable decrease in 5hMC (data not shown). To increase the number of enchondromas and to compare enchondromatosis and solitary tumours, we additionally evaluated the second cohort of enchondromatosis related cartilaginous tumours for 5mC and 5hmC levels. Levels of 5mC were significantly higher in solitary tumours compared to tumours occurring in the context of enchondromatosis (p = 0.012) (Fig. 4f), which was independent of histological grade. No difference was detectable for 5hmC (data not shown).

### Nuclear exclusion of TET1 is associated with loss of 5hmC in chondrosarcoma

Since we detected loss of 5-hmC in a subset of chondrosarcomas, which was not correlated with *IDH* mutation status, we evaluated whether, similar to gliomas [26], loss of 5-hmC was associated with nuclear exclusion of TET1. The majority of chondrosarcomas showed predominantly nuclear staining (N>C). No complete exclusion from the nucleus was seen in any of the chondrosarcomas (Fig. 5a). Chondrosarcomas with predominantly cytoplasmic expression of TET1 (C>N) showed significantly more often loss of 5hmC (p = 0.0001)(Fig. 5b).

## Discussion

We here report the analysis of mutations in genes encoding the metabolic enzymes IDH, SDH and FH in a cohort of ~100 central cartilaginous tumours, and demonstrate that the prevalence of mutations in *IDH1* or -*2* is ~60%, which is comparable to previously published data [8, 9]. As approximately 40% of the central chondrosarcomas lack detectable mutations in *IDH*, we investigated the possible involvement of two additional TCA cycle enzymes that are mutated in tumours, SDH and FH. Mutations in components of the SDH-complex and *FH* cause upregulation of succinate and fumarate, respectively, which, similar to d-2-HG, inhibit the TET enzymes [34–36] (Fig. 1). Using immunohistochemistry as a surrogate for mutation analysis, we show that *SDH* and *FH* mutations are not involved in the subset of chondrosarcomas that are wildtype for *IDH*. Moreover, it is unlikely that mutations in *TET2* are playing a prominent role in chondrosarcoma, since Tarpey et al. demonstrated *TET2* mutations in only one out of 49 (2%) chondrosarcomas subjected to whole-exome sequencing [47].

In contrast to gliomas, we here show that in chondrosarcoma, mutations in *IDH* are not significantly correlated with outcome. Disease specific survival and metastasis free survival did not differ between wild type and *IDH* mutant tumours of 63 patients. In a previous, separate series, we also found no prognostic value of these mutations [9]. Interestingly, patients with gliomas harboring *IDH* mutations have a more favorable outcome, independent of grade, as compared to gliomas that are wildtype for *IDH* [2, 14, 15]. In intrahepatic cholangiocarcinoma, as well as in leukemia, the prognostic significance of *IDH* mutations has remained controversial. The most recent studies however fail to demonstrate prognostic significance of the *IDH* mutation in these two tumour types [16, 17], which is comparable to chondrosarcoma.

Our aim was to evaluate the effect of *IDH* mutations on histone modifications, DNA modifications, chromatin remodeling, and subcellular localization of TET1 in a cohort of central cartilaginous tumours with known *IDH* mutation status. Immunoreactivity for the histone modification marks H3K4me3, H3K9me3 and H3K27me3 was observed in the majority of chondrosarcomas, irrespective of mutation status or histological grade. In contrast, we previously showed increased H3K9me3 in *SDH* mutant paragangliomas and *FH* mutant smooth muscle tumours using the same immunohistochemical methods. This was supported using SDH knockdown in cell lines, which demonstrates that our approach can detect differences in the methylation of these lysine residues [37]. Lu et al. showed increased H3K9me3 levels in *IDH1* mutant gliomas as compared to the *IDH* wildtype counterparts, which were almost negative for H3K9me3 [48]. Rohle et al. reported removal of the repressive H3K9me3 and H3K27me3 marks after inhibition of mutant IDH1 using AGI-5198 in *IDH1* mutant glioma cells [49]. In contrast to these findings in other tumour types, we showed previously that inhibition of mutant IDH1 in chondrosarcoma cell lines did not alter trimethylation of H3K4, H3K9 and H3K27 [50], which is in concordance with the present immunohistochemical results.

In addition to these covalent histone modifications, ATP-dependent chromatin remodeling complexes facilitate access of nucleosomal DNA. ATRX is an example of such a chromatin remodeler. We investigated expression of ATRX, since in gliomas loss of ATRX can be found in *IDH* mutant tumours [26]. Again, results are different from glioma as we found a low prevalence of loss of ATRX (5.6%), without any correlation to *IDH* mutation status in chondrosarcoma. The observed differences between *IDH* mutated chondrosarcomas versus *IDH* mutated gliomas on DNA and histone modifications, is likely attributable to additional genetic alterations that cooperate with mutant IDH to initiate cancer, e.g. *ATRX* and *TP53* mutations in *IDH* mutant gliomas and *COL2A1*, *YEATS2*, *NRAS*, *TP53*, Rb- and Hh- signaling mutations in chondrosarcomas [47, 51–53]. d-2-HG competitively inhibits the TET enzymes [18] which is expected to result in inhibition of the conversion of 5-mC to 5-hmC [54]. Thus, loss of 5hmC expression is expected in tumours harbouring mutations in *IDH*, *FH*, *SDH*, similar to leukemias with mutations in *TET2* [54]. Indeed, we previously showed loss of 5-hmC in *SDH* mutant paragangliomas and *FH* mutant smooth muscle tumours, again verifying our methodology [37]. Also in SDH deficient GIST, 5-hmC was low to absent [38]. For *IDH* mutant gliomas, however, reports have been conflicting [55, 56] and we now show that also in chondrosarcomas a correlation between mutations in *IDH* and loss of 5-hmC is absent. Despite this, we confirmed a correlation between loss of 5-hmC and diminished nuclear staining for TET1, which was also found in *IDH* wildtype gliomas [25] and SDH deficient paragangliomas [37].

Thus, overall we did not detect any differences in trimethylation of histone marks H3K4, -K9 and K27, and in 5-mC and 5-hmC levels between *IDH* mutant and *IDH* wildtype chondrosarcomas. Recently, Thienpont and colleagues reported that tumour hypoxia causes DNA hypermethylation and loss of 5hmC by reducing TET activity [57]. Interestingly, cartilage tissue and chondrosarcomas are known to have a hypoxic microenvironment [58, 59]; [60]; [61]. They also demonstrated a large overlap between genes hypermethylated in hypoxic versus *IDH1* mutant glioblastomas [57]. Thus, as tumour hypoxia may have the same effect as an *IDH* mutation in chondrosarcoma, this may explain why we did not detect any differences using immunohistochemistry.

Moreover, we previously demonstrated that mutant IDH1 is not essential for chondrosarcoma cell proliferation and survival, as its inhibition using AGI-5198 decreased levels of d-2-HG without affecting tumourigenic properties of chondrosarcoma cell lines [50]. We also showed that trimethylation of H3K4, H3K9 and H3K27 did not change using AGI-5198 [50]. On the other hand, we and others have shown that mutant IDH1 plays a crucial role in the early development of benign enchondromas, as osteoblast differentiation was inhibited while promoting chondrogenic differentiation of mesenchymal stem cells [62, 63]. Moreover, Jin et al. have shown that during this process the repressive mark H3K9me3 and the active mark H3K4me3 were increased [63]. Thus, taken together, these data suggest that *IDH* mutations, and the resulting epigenetic changes, are only important for the initiation of enchondroma. However, once these enchondromas have matured, and after progression to chondrosarcoma, other processes are likely involved, since detectable changes in histone marks or 5-hmC are lacking and there is no correlation between *IDH* mutation and prognosis in central cartilaginous tumours.

## Additional files

**Additional file 1: Table S1.** IDH primer sequences.

**Additional file 2: Figure S1.** No statistical significant difference was observed in disease specific survival between different *IDH* mutations (R132C, R132H, R132G, R132 l, R132S, R172S) compared to *IDH* wild-type chondrosarcomas (n = 63, p = 0.726).

**Additional file 3: Figure S2.** (A) All cartilage tumours on TMA were positive for SDHB, indicating absence of *SDH* mutations. (B) All cartilage tumours on TMA lacked detection of succinated protein using 2-SC staining, indicating absence of *FH* mutations, inset shows positive staining for 2-SC in a leiomyoma derived from a patient with a germline *FH* mutation as positive control (Scores were rounded to zero decimal places).

**Additional file 4: Table S2.** Mutation status for *SDH* (SDHB) and *FH* (2-SC) defined by immunohistochemistry on the TMA.
