# Supplementary material for: IDH1 or -2 mutations do not predict outcome and do not cause loss of 5-hydroxymethylcytosine or altered histone modifications in central chondrosarcomas
Source: Clin Sarcoma Res. 2017 May 4;7:8. doi: 10.1186/s13569-017-0074-6 (PMC5418698; doi:10.1186/s13569-017-0074-6)
Supplement: Supplementary file 1 — Additional file 1: Table S1. IDH primer sequences. [file 13569_2017_74_MOESM1_ESM.doc]

**IDH primer sequences**

IDH1 mutaties exon 4

R132

| primer name | M13 staart | seq | fragment size |  |
| --- | --- | --- | --- | --- |
| IDH1ex4 DNA forw | TGTAAAACGACGGCCAGT | CGGTCTTCAGAGAAGCCATT | 129 bp | identical to primer IDH1_ex4_c_f |
| IDH1ex4 DNA rev | CAGGAAACAGCTATGACC | gcaaaatcacattattgccaac |  |  |

R100

Primer name M13-staart seq

IDH1-R100-forw TGTAAAACGACGGCCAGT TGAGAAGAGGGTTGAGGAGT 139 bp

IDH1-R100-rev CAGGAAACAGCTATGACC TACCCATCCACTCACAAGCC

IDH2 mutaties exon 4

R140 en R172

|  | M13 | primer | fragment size |
| --- | --- | --- | --- |
| IDH2_ex4 forw | TGTAAAACGACGGCCAGT | GCTGCAGTGGGACCACTATT | 213 bp |
| IDH2_ex4 rev | CAGGAAACAGCTATGACC | CTCCACCCTGGCCTACCT |  |

| IDH2 R172 forw | TGTAAAACGACGGCCAGT | AGCCCATCATCTGCAAAAAC | 115 bp |
| --- | --- | --- | --- |
| IDH2 R172 rev | CAGGAAACAGCTATGACC | CAGTGGATCCCCTCTCCAC |  |
