# Supplementary figures and images for: IDH1 or -2 mutations do not predict outcome and do not cause loss of 5-hydroxymethylcytosine or altered histone modifications in central chondrosarcomas
Source: Clin Sarcoma Res. 2017 May 4;7:8. doi: 10.1186/s13569-017-0074-6 (PMC5418698; doi:10.1186/s13569-017-0074-6)

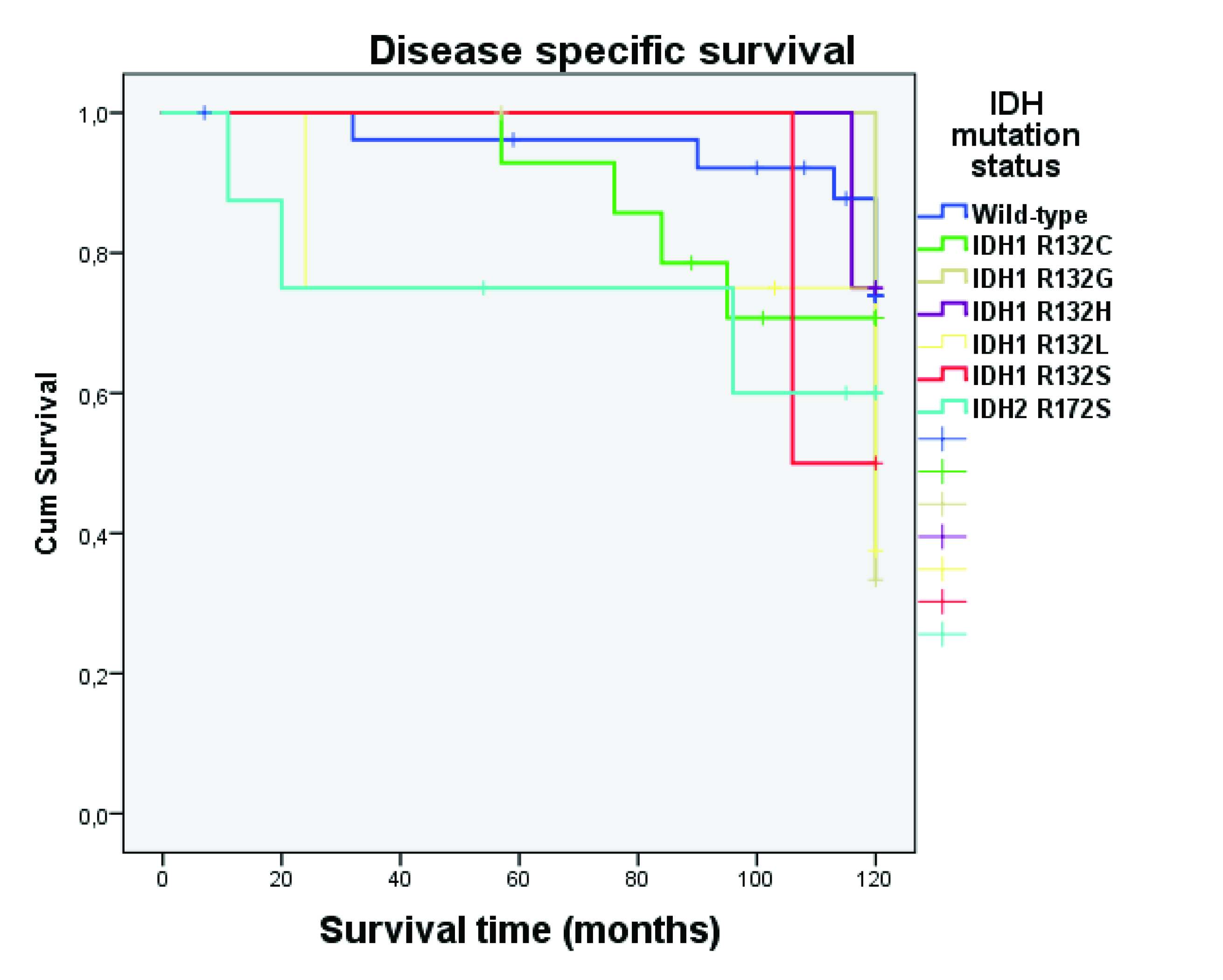

Supplement: Supplementary file 2 — Additional file 2: Figure S1. No statistical significant difference was observed in disease specific survival between different IDH mutations (R132C, R132H, R132G, R132 l, R132S, R172S) compared to IDH wild-type chondrosarcomas (n = 63, p = 0.726). [file 13569_2017_74_MOESM2_ESM.tif]

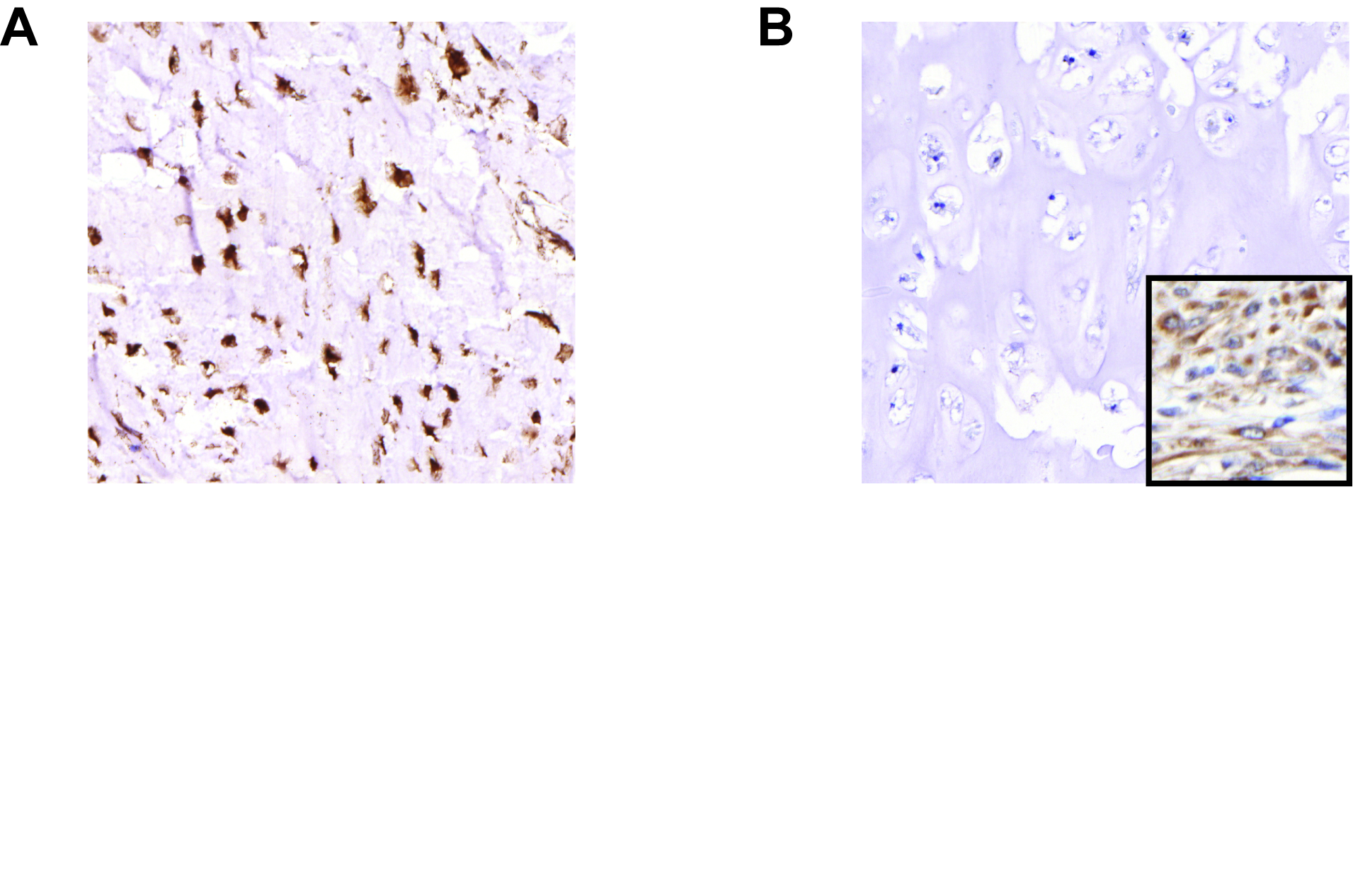

Supplement: Supplementary file 3 — Additional file 3: Figure S2. (A) All cartilage tumours on TMA were positive for SDHB, indicating absence of SDH mutations. (B) All cartilage tumours on TMA lacked detection of succinated protein using 2-SC staining, indicating absence of FH mutations, inset shows positive staining for 2-SC in a leiomyoma derived from a patient with a germline FH mutation as positive control (Scores were rounded to zero decimal places). [file 13569_2017_74_MOESM3_ESM.tif]
