# Supplementary material for: IDH1 or -2 mutations do not predict outcome and do not cause loss of 5-hydroxymethylcytosine or altered histone modifications in central chondrosarcomas
Source: Clin Sarcoma Res. 2017 May 4;7:8. doi: 10.1186/s13569-017-0074-6 (PMC5418698; doi:10.1186/s13569-017-0074-6)
Supplement: Supplementary file 4 — Additional file 4: Table S2. Mutation status for SDH (SDHB) and FH (2-SC) defined by immunohistochemistry on the TMA. [file 13569_2017_74_MOESM4_ESM.docx]

**Supplementary table 2: Mutation status for *SDH* (SDHB) and *FH* (2-SC) defined by immunohistochemistry on the TMA.**

|  | **Loss of SDHB** | **2-SC** |
| --- | --- | --- |
| **Enchondroma** | 0/8 | 0/6 |
| **ACT / grade I** | 0/67 | 0/45 |
| **Grade II** | 0/39 | 0/30 |
| **Grade III** | 0/17 | 0/14 |
